# Body and skull morphometric variations between two shovel-headed species of Amphisbaenia (Reptilia: Squamata) with morphofunctional inferences on burrowing

**DOI:** 10.7717/peerj.3581

**Published:** 2017-07-18

**Authors:** Leandro dos Santos Lima Hohl, Mariana Fiuza de Castro Loguercio, Fernando Lencastre Sicuro, José Duarte de Barros-Filho, Oscar Rocha-Barbosa

**Affiliations:** 1 Departamento de Zoologia, Instituto de Biologia Roberto Alcantara Gomes, Laboratório de Zoologia de Vertebrados Tetrapoda—LAZOVERTE, Universidade do Estado do Rio de Janeiro, Rio de Janeiro, Brazil; 2 Instituto de Biologia Roberto Alcantara Gomes, Programa de Pós-Graduação em Ecologia e Evolução, Universidade do Estado do Rio de Janeiro, Rio de Janeiro, Brazil; 3 Coordenação de Biologia, Centro Federal de Educação Tecnológica Celso Suckow da Fonseca—CEFET/RJ - Unidade Maracanã, Rio de Janeiro, Brazil; 4 Departamento de Ciências Fisiológicas, Instituto de Biologia Roberto Alcantara Gomes, BIOVASC, Universidade do Estado do Rio de Janeiro, Rio de Janeiro, Brazil

**Keywords:** Amphisbaenidae, Fossorial locomotion, Leposternon species, Locomotor performance, Morphological variation

## Abstract

**Background:**

Morphological descriptions comparing *Leposternon microcephalum* and *L. scutigerum* have been made previously. However, these taxa lack a formal quantitative morphological characterization, and comparative studies suggest that morphology and burrowing performance are be related. The excavatory movements of *L. microcephalum* have been described in detail. However, there is a lack of studies comparing locomotor patterns and/or performance among different amphisbaenids sharing the same skull shape. This paper presents the first study of comparative morphometric variations between two closely related amphisbaenid species, *L. microcephalum* and *L. scutigerum,* with functional inferences on fossorial locomotion efficiency.

**Methods:**

Inter-specific morphometric variations were verified through statistical analyses of body and cranial measures of *L. microcephalum* and *L. scutigerum* specimens. Their burrowing activity was assessed through X-ray videofluoroscopy and then compared. The influence of morphological variation on the speed of digging was tested among *Leposternon* individuals.

**Results:**

*Leposternon microcephalum* and *L. scutigerum* are morphometrically distinct species. The first is shorter and robust with a wider head while the other is more elongated and slim with a narrower head. They share the same excavatory movements. The animals analyzed reached relatively high speeds, but individuals with narrower skulls dug faster. A negative correlation between the speed and the width of skull was determined, but not with total length or diameter of the body.

**Discussion:**

The morphometric differences between *L. microcephalum* and *L. scutigerum* are in accord with morphological variations previously described. Since these species performed the same excavation pattern, we may infer that closely related amphisbaenids with the same skull type would exhibit the same excavatory pattern. The negative correlation between head width and excavation speed is also observed in others fossorial squamates. The robustness of the skull is also related to compression force in *L. microcephalum*. Individuals with wider heads are stronger. Thus, we suggest trade-offs between excavation speed and compression force during burrowing in this species.

## Introduction

Amphisbaenians are fossorial reptiles with an elongated and cylindrical body shape (Gans, 1969) and, except the three *Bipes* species, all (nearly 200 species, Uetz, Freed & Hosek, 2016) are limbless. They are a monophyletic group within Squamata (Gans, 1969; Kearney, 2003; Kearney & Stuart, 2004).

A strongly ossified and compact skull is common to all amphisbaenids. However, their heads may show four different morphological patterns: spade-head, keel-head, round-head, and shovel-head (Kearney, 2003). The “shovel” type, shared by the genera *Rhineura* (Rhineuridae, North America), *Dalophia* and *Monopeltis* (Amphisbaenidae, Africa), and *Leposternon* (Amphisbaenidae, South America), is considered the most specialized for digging (Gans, 1974; Gans, 2005; Kearney, 2003; Hohl et al., 2014). This means that these animals are able to penetrate more easily into highly compacted soils reaching greater depths.

Currently, there are ten recognized *Leposternon* species (Ribeiro et al., 2011; Ribeiro, Santos-Jr & Zaher, 2015). The species *Leposternon microcephalum* has a widespread distribution, occurring in different regions of Brazil, as well as in Bolivia, Paraguay, Argentina, and Uruguay (Perez & Ribeiro, 2008; Ribeiro et al., 2011). The widespread distribution of *L. microcephalum* throughout South America is associated with recognized geographic morphological variation (Barros-Filho, 2000; Gans & Montero, 2008), including Paraguayan individuals that were suggested as being a different species by Barros-Filho (2000). In the State of Rio de Janeiro, Brazil, *L. microcephalum* may occupy different soil types with different degrees of compaction and depth (Gonçalves-Dias & Barros-Filho, 1992). On the other hand, *L. scutigerum* has a more restricted distribution, endemic to the State of Rio de Janeiro, Brazil (Barros-Filho, 1994; Rocha et al., 2009), and occupies a specific soil type, less compacted and shallower than those occupied by *L. microcephalum* (Gonçalves-Dias & Barros-Filho, 1992). Also, it is included as Endangered (status EN) on the IUCN Red List of Threatened Species (Colli et al., 2016), and on Brazil’s National Red List (see Portaria MMA no 444, 17 December, 2014).

Morphological differences between individuals of *L. microcephalum* and *L. scutigerum* from the State of Rio de Janeiro include the number of annuli and vertebrae, snout-vent length, cephalic and pectoral shields configuration (Gans, 1971), and skull descriptive morphology (Barros-Filho, 2000; Gans & Montero, 2008). Among the osteological differences, the vertebrae numbers are 93–103 for *L. microcephalum* and 119–123 for *L. scutigerum* (Gans, 1971). Barros-Filho (2000) described the general anatomy of the skull of these species including features that we consider related to the excavation process. The skull of *L. microcephalum* can be distinguished from *L. scutigerum* by the diamond-shaped outline of the anterior view of the facial region; angulations between facial and medial skull regions tending to less than 120°(mean value  = 118.8°); the largest width of the facial region being conspicuously shorter than the width of the occipital region; the prefrontal bones not having any conspicuous lateral expansions; and the facial region not being especially expanded laterally at the transverse crest line (between facial and medial regions) (Barros-Filho, 2000). On the other hand, in *L. scutigerum*, the anterior view of the facial region has a triangular-shaped outline; the average angulation between facial and medial skull regions is 121.6°; the largest width of the facial region is very close to the width of the occipital region; the prefrontal bones present characteristic lateral expansions; and there is a characteristic lateral expansion at the transverse crest line (Barros-Filho, 2000).

Despite the good qualitative description of morphological variations present in the literature, there are no body and/or skull morphometric differences supported by statistical approaches for these species. Furthermore, the morphofunctional consequences of the morphological variations in *L. microcephalum* and *L. scutigerum* remain unknown.

Fossorial animals have to be able to travel along an existing tunnel, burrow or extend existing tunnel systems, and leave the surface by penetrating the soil (Gans, 1978). The high degree of specialization of the amphisbaenians is well expressed in the speed and effectiveness with which they burrow tunnel systems and penetrate the substrate, even in fairly hard soils (Gans, 1978).

The excavation patterns of the Amphisbaenia range from random movements of the head and lateral movements to “shovel” and “screw” movements, which vary according to the four head types (Gans, 1974). Shovel-headed amphisbaenians excavate through “shovel” movements of the head, which is considered the most specialized digging pattern (Gans, 1974). In fact, morphological features and underground locomotor performance seem to be related. For instance, body and head size and strength directly affect the ability of fossorial squamates to penetrate the substrate and move inside their galleries (Gans, 1974; Navas et al., 2004). Navas et al. (2004) postulated that *L. microcephalum* individuals with narrower heads dig faster than those with a wider head or more robust body. Gans (1978) suggested that the osteological reinforcement of the transverse crest, together with the discrete lateral projection of the otic capsules may be important for improving excavation efficiency. Furthermore, Vanzolini (1951) and Barros-Filho (1994) associated the fusion of cephalic and pectoral shields with the reduction of friction with the substrate, facilitating the underground displacement of the animal.

In the last 60 years, the locomotor behavior of *L. microcephalum* has been incidentally studied through visual observations (Kaiser, 1955), motion pictures of the external body (Navas et al., 2004), and the videofluoroscopy technique (Barros-Filho, Hohl & Rocha-Barbosa, 2008; Hohl et al., 2014). The most recent description of the excavatory cycle of *L. microcephalum* is presented as: (1) initial static position with the gular and anterior body regions lying over the tunnel floor; (2) retreating and downward bending of the head, with tip of snout touching the floor substrate; (3) a continuous upward and forward head movement, which compacts the substrate granules against the tunnel roof, while the pectoral region compresses the tunnel floor. This is followed by the dropping of the head, returning to the initial static position (Barros-Filho, Hohl & Rocha-Barbosa, 2008; Hohl et al., 2014).

Not only there are few studies of the underground locomotion of *L. microcephalum,* but all of them faced the problem of limited samples (Kaiser, 1955; Navas et al., 2004; Barros-Filho, Hohl & Rocha-Barbosa, 2008; Hohl et al., 2014). In fact, there are no studies of other species of *Leposternon*. Furthermore, there is a lack of studies comparing locomotor patterns and/or performance among different amphisbaenid species. Similarly, the review of literature shows that quantitative studies inferring the influence of morphological variation on the fossorial locomotor performance of amphisbaenids are scarce (e.g., López, Martín & Barbosa, 1997; Navas et al., 2004).

Given that there are notable morphological variations between *L. microcephalum* and *L. scutigerum*, we hypothesized that body and skull morphometric variation is also evident. Therefore, such variation could impact locomotor performance. Considering that *L. microcephalum* and *L. scutigerum* share the same general shovel-headed pattern, they should have similar excavatory movements. Furthermore, based on the observations of Navas et al. (2004) that individuals of *L. microcephalum* with narrower heads exert less force but dig faster than those with a wider head or more robust body, we believe that *Leposternon* individuals with narrower skulls and bodies may dig faster.

The main objective of the present study is to characterize the morphometric variations in body and skull between *L. microcephalum* and *L. scutigerum*, providing morphofunctional inferences on burrowing. We also describe the excavatory pattern and performance of *L. scutigerum*, comparing its locomotor traits with *L. microcephalum*; and we verify the relation between the length and robustness of the body and skull with the burrowing speed among *Leposternon* individuals.

## Material and Methods

### Morphometric analysis

A total of 26 adult *Leposternon* specimens were measured (*L. microcephalum* Wagler, 1824 *n* = 14; *L. scutigerum* (Hemprich, 1820) *n* = 12). The sample included eight live individuals used in the locomotion analyses and 18 specimens from scientific collections deposited at National Museum and Zoology Department of the Universidade Federal do Rio de Janeiro (UFRJ). The specimens from scientific collections were prepared, after we took the body measures, through exposure to *Dermestes* sp (Coleoptera, Insecta) larvae or maceration in hydrogen peroxide (H_2_O_2_) (see Barros-Filho, 2000). Individuals with a length of more than 150 mm were considered adults (Gans, 1971), and no sexual dimorphism was found for the variables analyzed. *Leposternon microcephalum* has some morphological variation along its geographic distribution in South America. Our analysis included only specimens from Rio de Janeiro, Brazil, therefore, we expected minor differences could exist considering particular ecotypes of this species. Identification of all specimens can be found in Table 1.

**Table 1:** Morphometric data of *L. microcephalum* (*n* = 14) and *L. scutigerum* (*n* = 12) specimens. All measures are in millimeters.

Specimens	Species	TL	D	CBL	MW	TCW	MWR
1/2012	*L. microcephalum*	360	13.7	20.8	12.5		
3/2012	*L. microcephalum*	333	15	22.3	9		
4/2012	*L. microcephalum*	294	16.2	16.2	12.5		
5/2012	*L. microcephalum*	354	16	18.7	9		
8/2012	*L. microcephalum*	370	17.5	22.3	12.3		
9/2012	*L. microcephalum*	364	20	20.5	13.7		
10/2012	*L. microcephalum*	298	16.2	18.5	10		
1/2017	*L. scutigerum*	428	11.7	14.8	6.5		
ZUFRJ240	*L. microcephalum*	260	13.1	14.3	8.2	5.1	5.5
ZUFRJ249	*L. microcephalum*	430	18.4	16.8	9.6	7	7.3
ZUFRJ285	*L. microcephalum*	463	20.6	18.9	11.1	7.3	8
ZUFRJ467	*L. microcephalum*	466	19.1	19.1	10.7	7.3	8.1
ZUFRJ468	*L. microcephalum*	326	10.5	14.2	7.9	4.9	5.2
ZUFRJ1320	*L. microcephalum*	490	16.4	20	12.7	7.5	8
ZUFRJ1321	*L. microcephalum*	425	17.6	18.8	10.2	7.2	7.7
MNRJ4036	*L. scutigerum*	481	12.4	17.5	9.8	8	8.5
MNRJ4037	*L. scutigerum*	489	15.3	17.9	9.4	7.8	8.1
MNRJ4038	*L. scutigerum*	408	10.4	14.7	7.4	5.9	5.9
MNRJ4458	*L. scutigerum*	490	12.5	17.8	9.7	8.2	8.3
MNRJ4490	*L. scutigerum*	390	10.7	14.1	7.4	5.6	5.6
MNRJ4791	*L. scutigerum*	443	10.7	16.8	8.8	7.3	7.3
ZUFRJ550	*L. scutigerum*	375	10.7	14.5	7.3	5.8	6.3
ZUFRJ1399	*L. scutigerum*	413	11.8	14.8	7.8	6	6.5
ZUFRJ1401	*L. scutigerum*	467	13.7	18	9.4	8.3	8.4
ZUFRJ1417	*L. scutigerum*	462	14.9	17.6	9.7	7.6	8
ZUFRJ1511	*L. scutigerum*	514	12.8	16.7	8.8	7.2	7.8

**Notes.**

Body measures TL total length D diameter

Skull measures CBL condylo-basal length MW maximum width TCW tranversal crest MWR maximum width of the rostral region

Scientific Collections MNRJ Museu Nacional da Universidade Federal do Rio de Janeiro ZUFRJ Zoologia, Universidade Federal do Rio de Janeiro

The first nine animals were recorded and released back into the wild and are identified by the number of specimen/year of recording.

**Figure 1:**
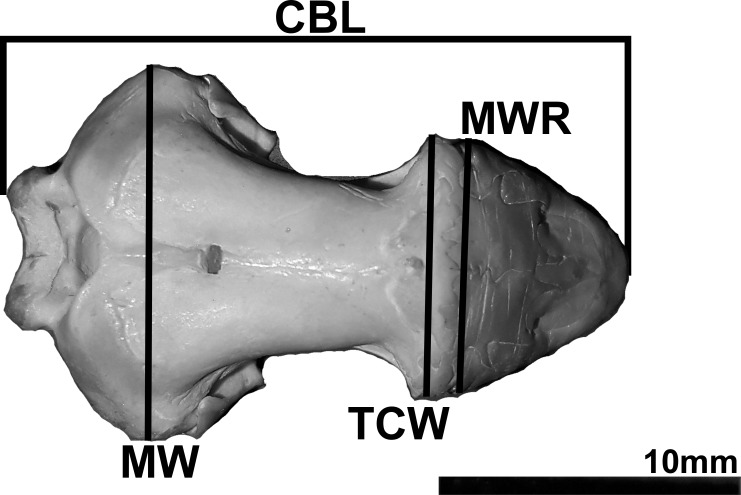
Photography of the dorsal view of a *Leposternon microcephalum* skull showing the measures taken. CBL = condylo-basal length; MW = maximum width; TCW = transverse crest width; MWR = maximum width of the rostral region.

Body measurements were taken using a measuring tape (in mm scale) whereas skull measurements were recorded with a digital caliper (model Pro-Max, Fowler-NSK scale 0.01 mm). The skull measurements were: condylo-basal length (CBL); maximum width (MW), situated at the occipital region covering the optical capsules; the transverse crest width (TCW); and maximum width of the rostral region (MWR) (Fig. 1). The body measurements included: the total body length (TL) and diameter (D) in the middle of the body. The terminology of cranial structures follows Gans & Montero (2008). The skull measurements of the live individuals were taken using X-ray records through the software Tracker v. 4.96. The distance between lead pieces in the wall of the terrarium was used as scale (5 cm). For this sample, it was not possible to measure the TCW and MWR.

The frequency distributions of the morphometric variables were tested for normality (Shapiro–Wilk’s T) and homoscedasticity (Levene), as well as, skewness and kurtosis. The Wilcoxon-Mann-Whitney *U*-Tests were used to test intra- and inter-specific morphometric variations among *L. scutigerum* and *L. microcephalum* specimens by comparing body and skull measurements. Comparisons between groups were performed through Principal Component Analysis (PCA) as a data exploratory method, over the standardized values of the original variables considered (TL, D, CBL and MW), to verify their trends of variation and the group distribution in their multivariate morphological space. Statistical analyses were performed with Statistica v.8.0 (StatSoft, Inc., Tulsa, OK, USA).

### Locomotion analysis

Eight adult individuals of the genus *Leposternon* from Rio de Janeiro City were analyzed (*L. microcephalum n* = 7; *L. scutigerum n* = 1). *Leposternon scutigerum* is an elusive species. The individuals of *L. microcephalum* were collected, recorded and analyzed by Hohl et al. (2014). Field collections were approved by the Instituto Chico Mendes de Conservação da Biodiversidade (ICMBio), environmental agency of the Brazilian government, with a permanent license number for collecting zoological material (15337), since May 28th, 2008. This locomotor behavior study was approved by the Ethics Committee for the Care and Use of Experimental Animals of the Instituto de Biologia Roberto Alcantara Gomes (CEUA/IBRAG/015/2017). The individuals are identified by number of specimen/year of recording in Table 1.

Locomotion was recorded using videofluoroscopy. This technique, based on X-ray recording, has been used in behavioral studies of fossorial species of amphisbaenians (Barros-Filho, Hohl & Rocha-Barbosa, 2008; Hohl et al., 2014) and caecilians (Summers & O’Reilly, 1997; Measey & Herrel, 2006; Herrel & Measey, 2010; Herrel & Measey, 2012).

Before recording, *L. scutigerum* was maintained in the laboratory at the Laboratório de Zoologia de Vertebrados-Tetrapoda (LAZOVERTE), Universidade do Estado do Rio de Janeiro (UERJ) under the same conditions presented by Hohl et al. (2014), i.e., kept in a plastic box containing humid humus-rich soil, and feed with earthworms once a week. The X-rays were performed at the Hospital Universitário Pedro Ernesto (HUPE/UERJ), Rio de Janeiro, with a Toshiba videofluoroscopy machine (Toshiba Ultimax from Toshiba Medical Systems) that films at 30 frames per second with calibration of 200 mA and 40 kV. In addition, following the same procedures used by Hohl et al. (2014) to film individuals of *L. microcephalum*, *L. scutigerum* was kept in a glass terrarium filled with dry/loose semolina, and with lead markers placed on the outer face of the wall terrarium. More details about the recording procedure can be found in Hohl et al. (2014). After filming, the animal was released back into the wild.

A total recording time of 7 min and 40 s containing 32 motion sequences of *L. scutigerum* was analyzed frame by frame using the Toshiba Daicom Viewer software. The excavatory pattern was analyzed taking into account the details of the body postures and movements performed. The locomotor performance was evaluated through the distance covered, duration of the movement, speed and frequency of cycles performed, according to Hohl et al. (2014).

Despite all *Leposternon* individuals sharing the same skull type, “shovel”, the sample contained two species. Thus, the excavatory pattern of *L. scutigerum* (*n* = 37 cycles) was determined and compared inter-specifically with the three-step gait pattern exhibited by *L. microcephalum* (*n* = 132 cycles), according to Barros-Filho, Hohl & Rocha-Barbosa (2008) and Hohl et al. (2014).

The small sample of individuals hindered more comprehensive inter-specific statistical comparisons. Therefore, we evaluated the degree of variation observed in the video footage among the individuals recorded as a starting point to estimate possible differences between locomotor performance in *L. scutigerum* and *L. microcephalum*. The three fastest excavatory cycles (i.e., in which the animals reached highest speeds) performed by each individual (*n* = 7 *L. microcephalum* and *n* = 21 excavatory cycles; *n* = 1 *L. scutigerum* and *n* = 3 excavatory cycles) were quantified according to the locomotory parameters proposed.

### Test of the relation between morphometric variables and burrowing speed

The influence of morphological variations on locomotor performance was assessed through Multiple Linear Regressions. The speed, expressing the degree of tunneling specialization (Gans, 1978), was used as the dependent variable to be regressed against body and skull measurements considered important for excavation ability according to Gans (1974) and Navas et al. (2004): total body length and diameter, and the maximum width of skull (independent variables). The analyses were performed with Statistica v.8.0 (StatSoft, Inc., Tulsa, OK, USA) first adding only *L. microcephalum* individuals, and then inserting the one individual of *L. scutigerum*.

## Results

### Morphological variation

Inter-specific differences between *L. microcephalum* and *L. scutigerum* were observed in total length (TL) (*U* = 33.5, *p* < 0.01), diameter of the body (D) (*U* = 17.5, *p* < 0.001), condylo-basal length (CBL) (*U* = 34.5, *p* < 0.01), and maximum width of the skull (MW) (*U* = 27.0, *p* < 0.01). However, there were no statistical differences in the transverse crest width (TCW) or maximum width of the rostral region of the skull (MWR). Statistics are given in Table 2.

**Table 2:** Comparative analyses of body and skull morphometric data between *L. scutigerum* and *L. microcephalum*.

	*L. microcephalum*	*L. scutigerum*	C. V. (%) Lm/Ls	*U*-Test *P* value
TL	371.1 ± 69.1 (*N* = 15)	446.6 ± 44.1 (*N* = 12)	18.6 / 9.9	<0.01**
D	16.45 ± 2.77 (*N* = 14)	12.31 ± 1.65 (*N* = 12)	16.8 / 13.4	<0.001**
CBL	18.67 ± 2.56 (*N* = 14)	16.27 ± 1.55 (*N* = 12)	13.7 / 9.5	<0.01**
MW	10.67 ± 1.84 (*N* = 14)	8.5 ± 1.16 (*N* = 12)	17.2 / 13.6	<0.01**
TCW	6.61 ± 1.11 (*N* = 7)	7.06 ± 1.04 (*N* = 11)	16.8 / 14.7	>0.05 ns
MWR	7.11 ± 1.24 (*N* = 7)	7.34 ± 1.07 (*N* = 11)	17.4 / 14.6	>0.05 ns

**Notes.**

C. V. Coefficient of Variation Lm *Leposternon microcephalum* Ls *Leposternon scutigerum*

Values are presented by means and standard deviation, in millimeters.

**Figure 2:**
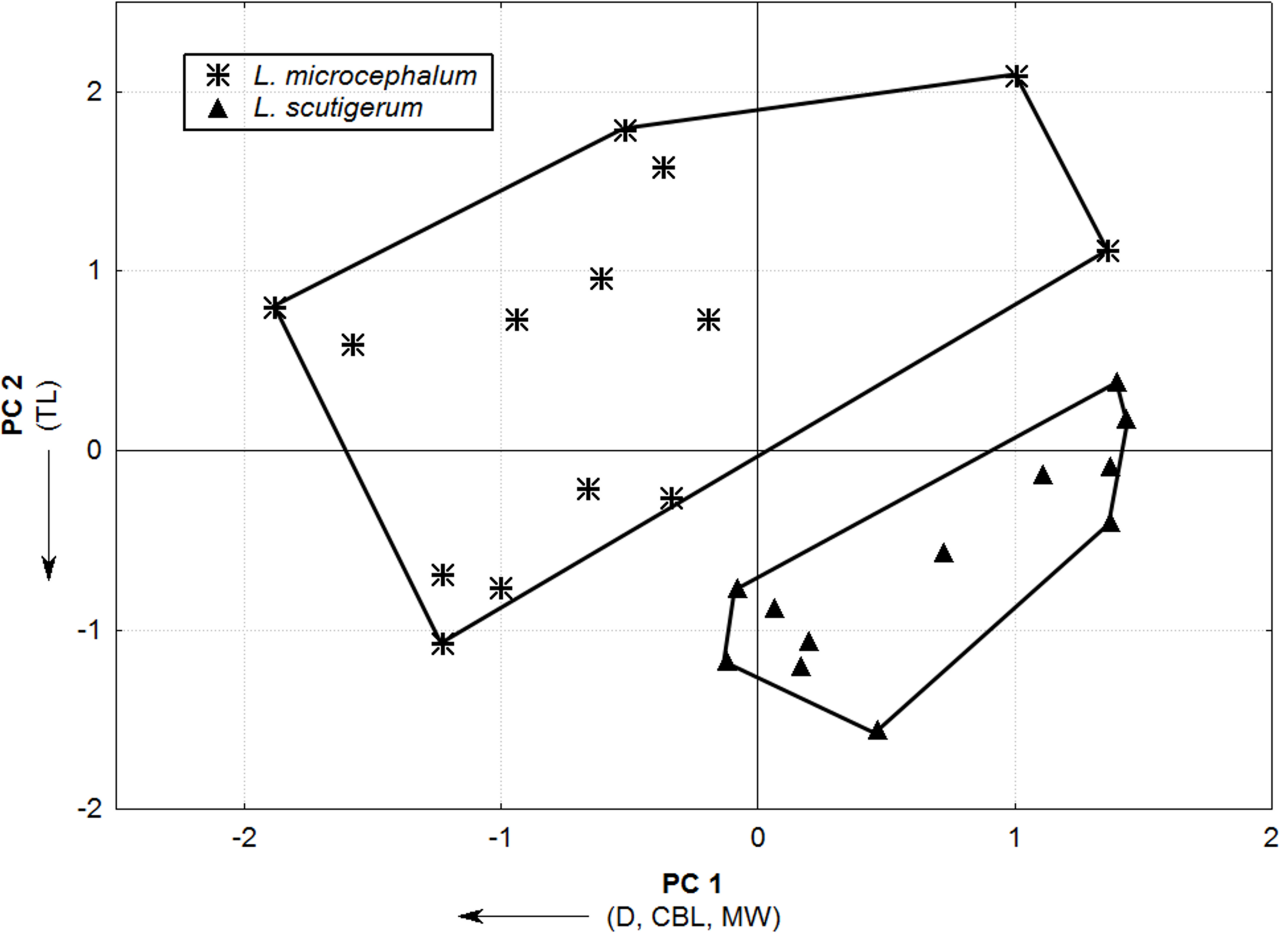
Principal Component Analysis scatterplot showing the distribution of *L. microcephalum* and *L. scutigerum* specimens on the multivariate space through the two main axis of variation (PC1 and PC2). Arrows indicate the direction of the correlation of each variable with the different PCs.

The PCA analysis indicated two main axes of group differentiation: PC1 and PC2 explained, respectively, 60.63% and 25.03% of the variation. On PC1, the variables D, CBL, and MW loaded strongly and negatively. On PC2, the total length of the body loaded negatively and strongly (Table 3). According to the PCA scatterplot (Fig. 2), *L. scutigerum* specimens occupied higher positive scores on PC1 and negative scores on PC2, tending to exhibit a greater elongated and slim body shape with a narrower head. On the other hand, *L. microcephalum* specimens occupied greater negative scores on PC1 and positive scores on PC2, featuring a shorter and robust body shape with a wider head.

**Table 3:** The PCA loadings of the variables for the two main axis of variation (PC1 and PC2). Larger absolute values indicate more influential traits on the multivariate space. The higher loadings in each axis were highlighted in bold. Eigenvalues and cumulative percentage of variation explained are also indicated.

	PC 1	PC 2
TL	−0.06223	**−0.99702**
D	**−0.88608**	0.03308
CBL	**−0.89178**	−0.04454
MW	**−0.91613**	0.07889
Eigenvalue	2.423571	1.000637
Cumulative %	60.6308	85.6638

### Locomotor variation

A detailed analysis of the movement, through visual observation of X-ray images, revealed that the three-step excavatory cycle of *L. scutigerum* seemed to be the same as *L. microcephalum*. Barros-Filho, Hohl & Rocha-Barbosa (2008) and Hohl et al. (2014) described this movement as: (1) initial static position with the gular and anterior body regions lying over the tunnel floor; (2) retreating and downward bending of the head, with tip of snout touching the floor substrate; (3) a continuous upward and forwardhead movement, which compacts the substrate granules against the tunnel roof, while the pectoral region compresses the tunnel floor. This is followed by the dropping of the head, returning to the initial static position.

According to the locomotor performance data, *L. scutigerum* constructed galleries with an average speed of 0.465 cm s^−1^ (±0.2), travelling 0.58 cm (±0.15) in 1.26 s (±0.43) seconds, and performed almost a complete excavatory cycle each second (0.8 ± 0.3 Hz). The three fastest excavatory cycles of *L. scutigerum* showed that it constructed galleries with an average speed of 0.757 cm s^−1^ (±0.06), travelling 0.73 cm (±0.11) in 0.83 s (±0.23) seconds, and performed a complete excavatory cycle each second (1.06 ± 0.17 Hz). During the three fastest excavatory cycles, *L. microcephalum* was able to reach an average speed of 0.350 cm s^−1^ (±0.09), travelling 0.59 cm (±0.18) in 1.77 s (±0.59), and performed about a half complete excavatory cycle each second (0.61 ±0.15 Hz).

The mean values and SDs of the locomotor performance variables suggest that some inter-specific differences may exist between the species. *Leposternon scutigerum* presented higher averages for speed, travel distance and frequency of excavatory cycles, and lower values for cycle duration in relation to those found for *L. microcephalum*. On the other hand, the values of the coefficients of variation of the locomotor variables indicated some constancy in the species’ performances along the intra-specific footages. Therefore, despite the sample size limitation, based on this functional approach to the excavatory performance of the species, we hypothesized that *L. scutigerum* could be a better excavator than *L. microcephalum,* in the sense of speed of soil penetration and gallery construction. Descriptive statistics values are presented in Table 4.

**Table 4:** Descriptive statistical values of the fastest excavatory cycles performed by *L. scutigerum* (three cycles of one individual) and *L. microcephalum* (21 cycles of seven individuals).

	*L. microcephalum*	*L. scutigerum*	C. V. (%) Lm/Ls
Speed (cm s^−1^)	0.350 ± 0.09	0.757 ± 0.06	25.8 / 8
Distance (cm)	0.59 ± 0.18	0.73 ± 0.11	30.6 / 15.4
Time (s)	1.77 ± 0.59	0.83 ± 0.23	33.3 / 28
Frequency (Hz)	0.61 ± 0.15	1.06 ± 0.17	24.2 / 16.6

**Notes.**

C. V. Coefficient of Variation Lm *Leposternon microcephalum* Ls *Leposternon scutigerum*

Values are presented by means and standard deviation.

### Test of the relation between morphometric variables and burrowing speed

**Figure 3:**
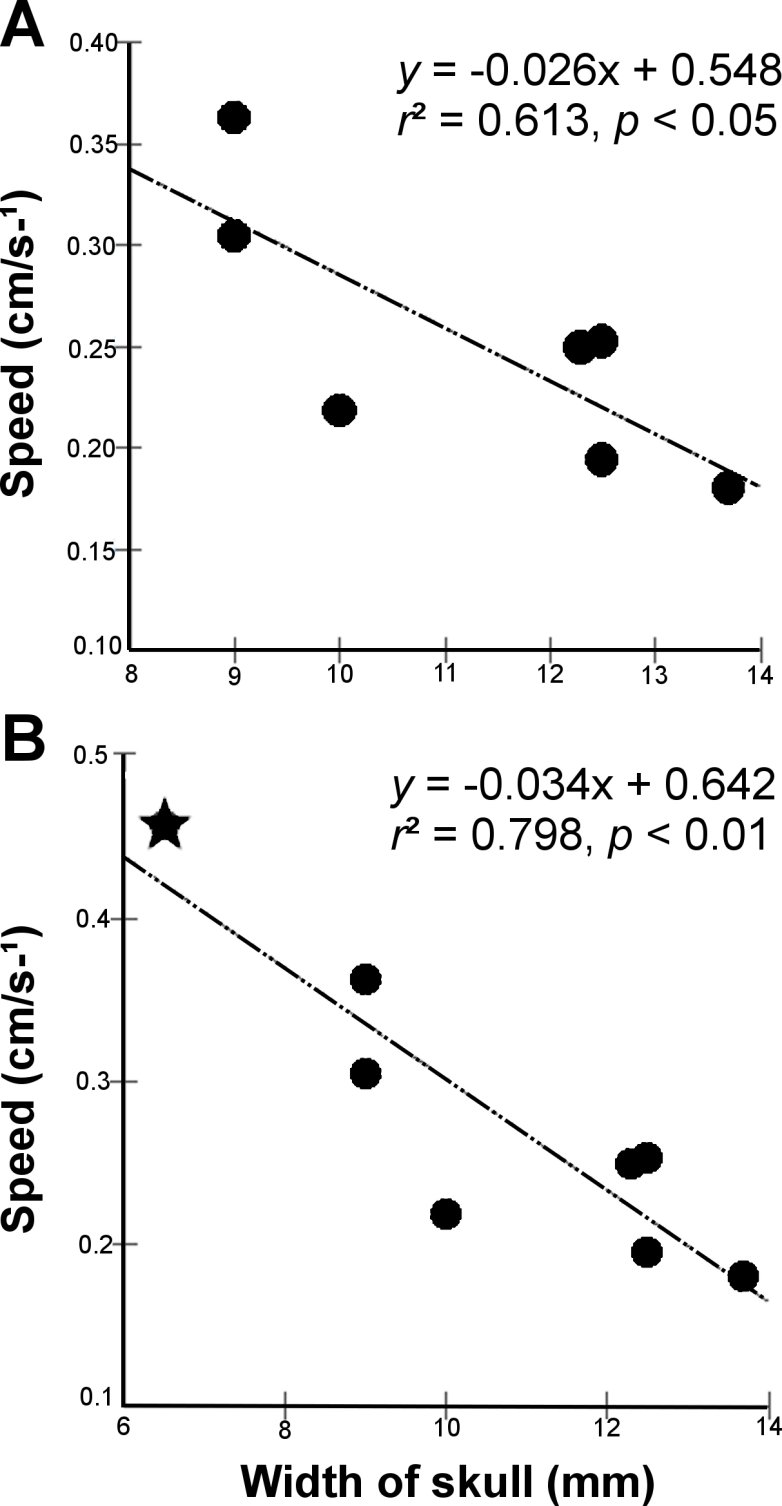
Excavatory cycle speed as a function of width of skull in *Leposternon* individuals. (A) Analysis restricted to *L. microcephalum*. (B) Analysis after insertion of *L. scutigerum*, represented by the star.

When the analysis was restricted to *L. microcephalum* individuals, linear regression demonstrated a negative correlation between the speed (dependent variable) and the width of skull (independent variable) (*r*^2^ = 0.613; *p* = 0.037) (Fig. 3A), but not with total length (*r*^2^ = 0.001; *p* = 0.944) or diameter of the body (*r*^2^ = 0.08; *p* = 0.54). When inserting the unique specimen of *L. scutigerum,* the analysis revealed a stronger and significant negative correlation between speed and width of skull (*r*^2^ = 0.798; *p* = 0.003) Fig. 3B. Relations with total length (*r*^2^ = 0.30; *p* = 0.153) or diameter of the body (*r*^2^ = 0.42; *p* = 0.079) remained non-significant.

## Discussion

### Inter-specific morphometric variation

Anatomical differences between *L. microcephalum* and *L. scutigerum* from Rio de Janeiro State are well known (Gans, 1971; Barros-Filho, 2000; Gans & Montero, 2008). The initial hypothesis that body and skull morphometric differences exist was corroborated. Significant differences between these two species in total length and diameter of the body, and maximum width of skull exist.

As suggested by the great number of vertebrae, *L. microcephalum* with 93–103 vertebrae and *L. scutigerum* with 119–123 (Gans, 1971), *L. scutigerum* presented a more elongated body. According to Barros-Filho (2000), the largest width of the facial region also covering the transverse crest of *L. scutigerum* is very close to the maximum width of the skull at the occipital region. This configuration is different for *L. microcephalum*, in which the largest width of the facial region is shorter than the width of the occipital region. Based on this difference, that author concluded that *L. scutigerum* “has a very characteristic skull, specially the lateral expansion of the facial region, at the transverse crest line”. However, our results indicated statistical differences between these species for maximum width of skull (occipital region), but not for transverse crest width or maximum width of the rostralregion (facial region). Thus, the allometric relation between facial and occipital regions of these species proposed by Barros-Filho (2000) is more related to occipital differences than facial ones.

### Inter-specific comparisons of excavatory behavior

Barros-Filho, Hohl & Rocha-Barbosa (2008) discussed the efficiency of videofluoroscopy as a methodology for analyzing locomotor behavior in fossorial squamates. They concluded that among the methodologies previously used (e.g., visual observations and motion pictures of the external body), videofluoroscopy was the most efficient in analyzing excavatory cycles. Considering that the present study also used videofluoroscopy as the methodology to access the fossorial locomotion of *L. scutigerum*, and replicated the movie set used by Barros-Filho, Hohl & Rocha-Barbosa (2008) and Hohl et al. (2014) (i.e., the same glass terrarium marked with lead pieces and filled with dry/loose semolina), we conclude that the comparison results of locomotor pattern and performance between *L. scutigerum* and *L. microcephalum* were not influenced by methodological factors.

*Leposternon scutigerum* performed, in horizontal and vertical directions, the same three-step excavatory cycle first described by Barros-Filho, Hohl & Rocha-Barbosa (2008) for *L. microcephalum*, reinforced by Hohl et al. (2014), as a retreating and downward bending of the head from an initial static position followed by an upward and forward head movement. This result suggests some partial support for the initial hypothesis that morphologically similar amphisbaenids would exhibit the same excavatory pattern. The results also suggests some support for the assumption of Barros-Filho, Hohl & Rocha-Barbosa (2008) that, in spite of specific differences among the shovel-headed amphisbaenid species, the excavatory pattern was the same for all of those species, based on the fact that the shovel-head is, apparently, a convergence among the group (cf. Gans, 1974).

However, despite sharing the same skull type and performing three-step excavatory pattern, our quantitative approach of locomotor performance of *L. scutigerum* and *L. microcephalum* suggested differences in all parameters analyzed (travel distance, excavatory cycle duration, speed, and frequency). According to Hohl et al. (2014), *L. microcephalum* was able to build a gallery of 12 cm in length in 1 min. Our results showed that this species was able to build a gallery of 21.6 cm in length in 1 min (mean values based on the three fastest cycles), whereas the single individual of *L. scutigerum* studied was able to build a gallery of 46.4 cm in length in 1 min, i.e., more than twice, on average. Despite the lack of a formal statistical significance test, these values suggest that *L. scutigerum* may be a faster digger than *L. microcephalum*.

### Morphological features and locomotor performance

Gans (1974) and Navas et al. (2004) postulated that body, head size and strength directly affect the speed of fossorial squamates to penetrate the substrate and move inside their galleries. However, they only reported it qualitatively. Navas et al. (2004), although relating the robustness of the head with the compression force, did not obtain the speed with which the animals excavated to associate with head width. According to our quantitative approach these previous statements seem partially true. Body dimensions (total length or diameter) seem to be not related to the increased speed of excavation. Despite the *p*-value above the alpha = 0.05, it seems to be a trend of correlation between body diameter and speed. We are positive that a larger sample could bring more assurance to this hypothesis. However, regression analysis showed that the width of skull is related to the speed of excavation (individuals with slender skulls seem to be faster). The finding, that head width is negatively correlated with excavation speed, is in agreement with previous studies. López, Martín & Barbosa (1997) showed that individuals of *Blanus cinereus* with narrower, longer heads burrow faster. Vanhooydonck et al. (2011) showed that in burrowing skinks *Acontias percivali*, individuals with narrow heads were able to dig faster than broader-headed ones. Similar to our results, Vanhooydonck et al. (2011) also concluded that the speed in soil penetration is only predicted by the head width, curiouslly getting similar statistical values (*L. microcephalum - r*^2^ = 0.613, *p* = 0.037; *Acontias percivali* - *r*^2^ = 0.64, *p* = 0.03).

The robustness of the skull has been demonstrated to be an important characteristic to improve soil penetration. Beyond being associated with excavation speed, as here presented, Navas et al. (2004) also demonstrated that the robustness of the skull is also related to compression force among *L. microcephalum*. Individuals with wider heads produced greater compression forces (about 25 N). Likewise, Vanhooydonck et al. (2011) and Baeckens et al. (2016) showed a strong positive relation between head size and bite force in *A. percivali* and *Trogonophis wiemanni*, respectively. According to these authors, bite performance also increased with head size in these species.

Besides individuals of the *L. microcephalum* species, our sample contained a unique *L. scutigerum* specimen that presented the narrowest skull (6.5 mm), and, according to our results, it showed the highest mean speed. Comparing inter-specifically, the morphological features of *L. scutigerum* supported the results of the locomotor performance analysis, and the differences in relation to *L. microcephalum*.

Gans (1978) highlighted that the slight lateral protrusion of the otic capsules is important for improving the excavation efficiency, here corroborated by the negative correlation between the width of skull and speed through regression analysis. According to our morphological results based on a larger sample, the species *L. scutigerum* presented a narrower skull (maximum width situated at the occipital region covering the otic capsules) when compared to *L. microcephalum* which possesses a wider skull.

Future studies on compression force and bite performance are needed for these species. Although *L. microcephalum* exhibited low speed during digging in relation to *L. scutigerum*, according to Navas et al. (2004), Vanhooydonck et al. (2011), and Baeckens et al. (2016), it might be able to exert greater compression and bite forces. Even small variations in head width may present a large impact on burrowing compression force (Navas et al., 2004) and speed (Vanhooydonck et al., 2011). Thus, our present data on speed, associated with the data from Navas et al. (2004) on compression forces suggest a performance trade-off between compression forces and burrowing speeds in the shovel-headed *Leposternon* (i.e., individuals with narrow heads excavate rapidly but exert less push force, while individuals with robust heads excavate slowly but exert more push force). This relation may be associated with the ecology and geographic distribution differences among *L. microcephalum* (larger geographic distribution occupying different soil types) and *L. scutigerum* (limited distribution occupying a restricted soil type).

## Additional Information and Declarations

### Field Study Permissions

The following information was supplied relating to field study approvals (i.e., approving body and any reference numbers): Field experiments were approved by the Instituto Chico Mendes de Conservação da Biodiversidade (ICMBio), environmental agency of the Brazilian government.

### Data Availability

The following information was supplied regarding data availability: The raw data is included in Table 1.
